# Supplementary material for: Clinical cancer genomic profiling by three-platform sequencing of whole genome, whole exome and transcriptome
Source: Nat Commun. 2018 Sep 27;9:3962. doi: 10.1038/s41467-018-06485-7 (PMC6160438; doi:10.1038/s41467-018-06485-7)
Supplement: Supplementary file 1 — Supplementary Information [file 41467_2018_6485_MOESM1_ESM.pdf]

Clinical Cancer Genomic Profiling by Three-Platform Sequencing of Whole Genome, Whole Exome and Transcriptome

Rusch, et al.

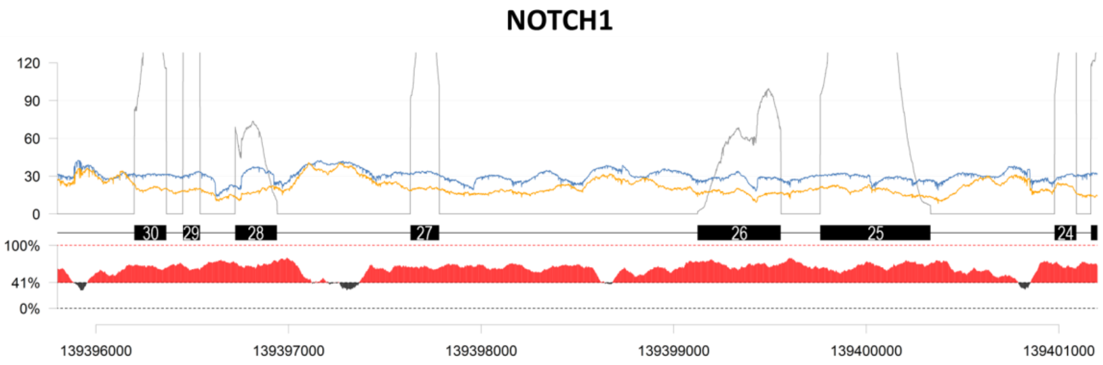

Supplementary Figure 1. Comparison of Coverage by WGS and WES in NOTCH1. The orange and blue curves show mean WGS coverage across germline samples sequenced using PCR-based (N=8) and PCR-free (N=78) protocols, respectively. The gray curve depicts WES coverage over coding exons only. The gene model is depicted below, with thick black boxes indicating coding exons. GC content is rendered below (100 bp window with moving step of 1bp), colored red where GC% is above the genome-wide average of 41%, and black where below. The genomic region shown includes exons 24-30 of *NOTCH1* and shows the improved coverage of PCR-free WGS.

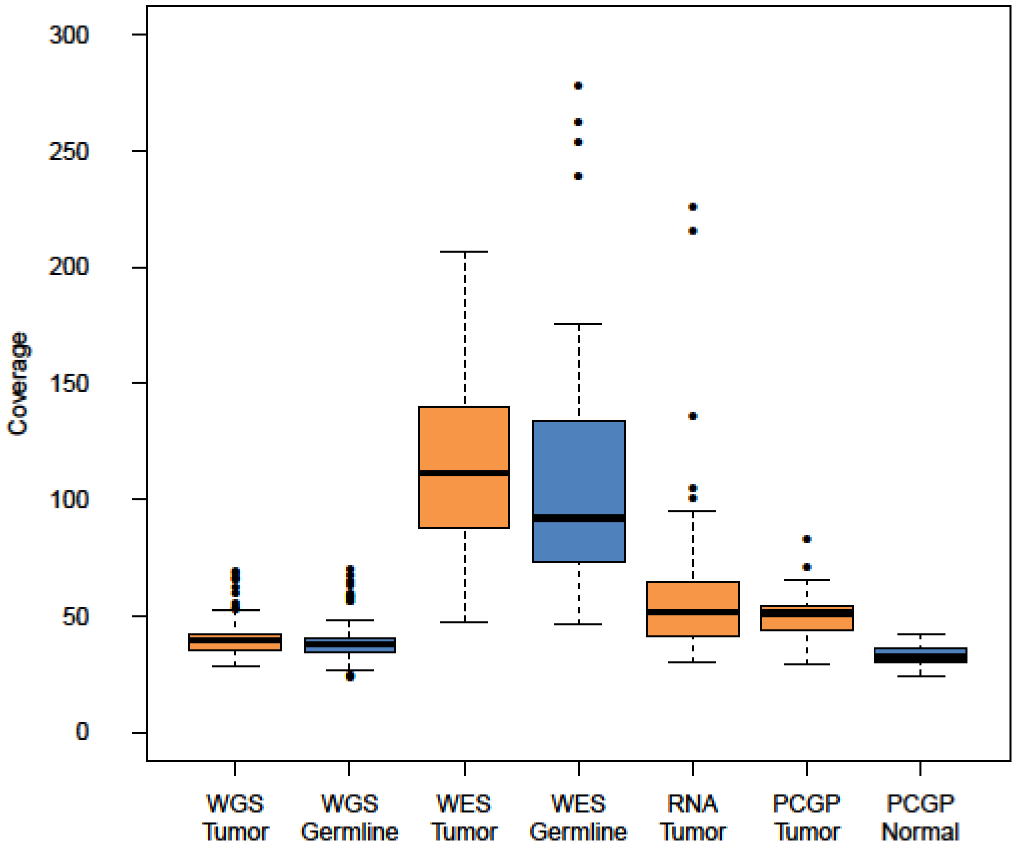

Supplementary Figure 2. Sequence coverage of the three platforms used for three-platform sequencing. Mean coverage of the 5 sequencing experiments for each of the 78 cases in the clinical study are shown in the first 5 columns. Coverage is averaged over the entire genome for WGS and across bases in the coding exome for WES and RNA-Seq. The right two columns show WGS coverage of the 33 overlapping PCGP cases for comparison. The dark lines indicate median values. The boxes indicate the interquartile range (IQR). Whiskers extend to 1.5×*IQR* unless there are no outliers that exceed 1.5×*IQR*, in which case they are the minimum and maximum values.

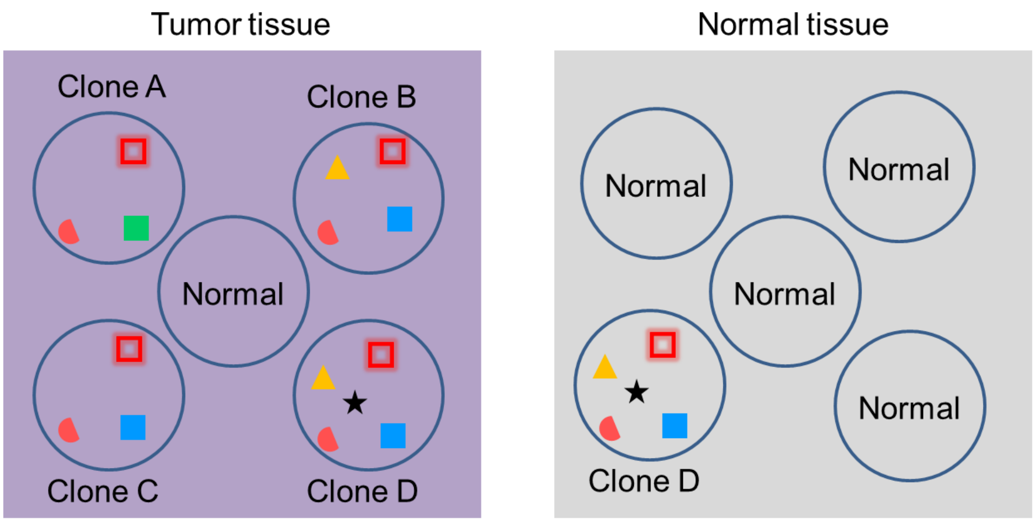

Mutation	Clonality	Detectable	Reason	Example
◖ □	Clonal	Yes	Higher MAF in tumor than in normal	H3F3A
▲ ■	Subclonal	Likely	Higher MAF in tumor than in normal	GPR26
■	Subclonal	Yes	Absent in normal	BDP1
★	Subclonal	No	MAF comparable in tumor and normal	NPFFR2

Supplementary Figure 3. Tumor in Normal Contamination of SJHGG001. This illustrative model depicts 4 tumor clones and contaminating normal cells in the tumor sample of SJHGG001 (left), and normal cells with contaminating tumor cells from a single clone in the normal sample (right). Various profiles of mutations are depicted by symbols and described in the table below along with details of their detectability.

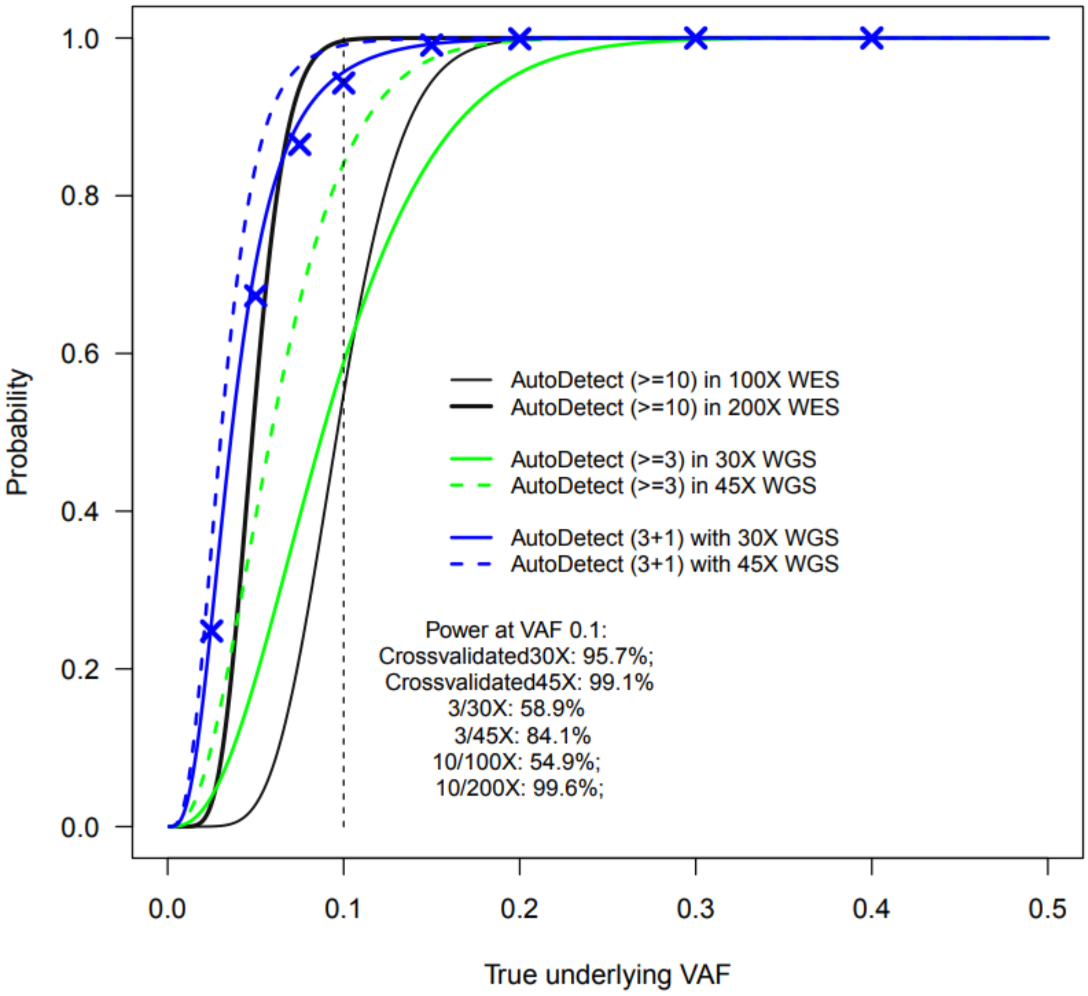

Supplementary Figure 4. Limit of detection analysis. The probability of detecting a variant with sufficient read evidence to make a call in WES and WGS is shown in black and green lines. We chose 100X WES and 30X WGS for our current study, 200X WES based on prior literature [1], and 45X WGS based on improvements made to our clinical genomics program after this pilot study completed. The blue lines show the probability of detection by at least 3 reads in WGS or WES and at least one read in the other platform, which is the standard used by the variant detection pipeline and cross-validation filtering pipeline, respectively. Details of the calculations are in the Supplementary Methods section.

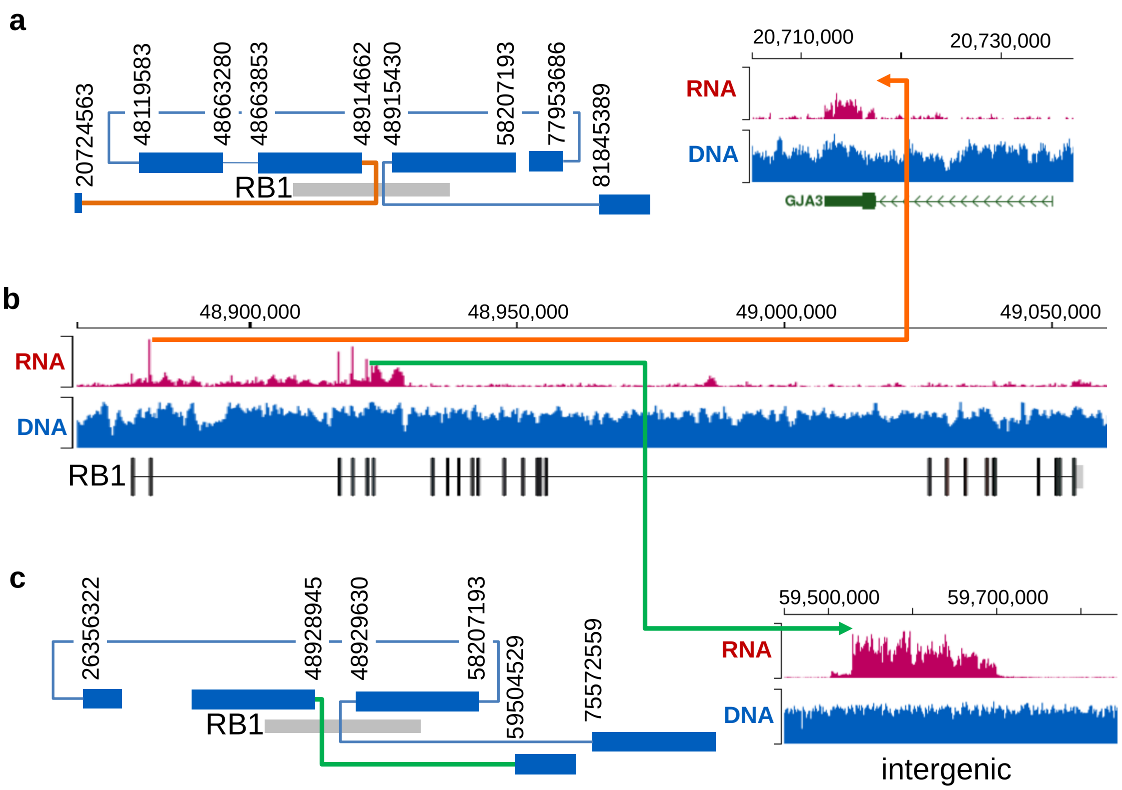

Supplementary Figure 5. Bi-allelic loss of RB1 in a retinoblastoma caused by two chromothripsis events. A total of 70 CNAs and 41 SVs (including two *RB1* fusions) were detected on chromosome 13. The *RB1* gene has normal ploidy except for two <1Kb intronic deletions, one of 781bp in intron 2 and another of 686bp in intron 6. Lines connecting genomic positions indicate a DNA SV. The wiggle plot marked DNA and RNA shows the read-depth in WGS and RNA-Seq of the tumor genome, respectively. **(a)** Complex re-arrangement at intron 2 of *RB1*. The orange line shows that one of the intron 2 SVs is linked to the *GJA3* gene located 28Mb upstream, resulting in an out-of-frame fusion transcript that links *RB1* exon 2 to *GJA3* 5' UTR (indicated by the orange arrow). **(b)** WGS and RNA-Seq at the *RB1* locus. Orange and green lines in RNA-Seq link exons 2 and 6 of *RB1* to their respective fusion partners. **(c)** Complex re-arrangement at intron 6 of *RB1*. The green line shows that one of the intron 6 SVs is linked to an intergenic region 10Mb downstream, resulting in a fusion transcript linking *RB1* exon 6 to aberrant transcription in the intergenic region, causing truncation of the *RB1* protein.

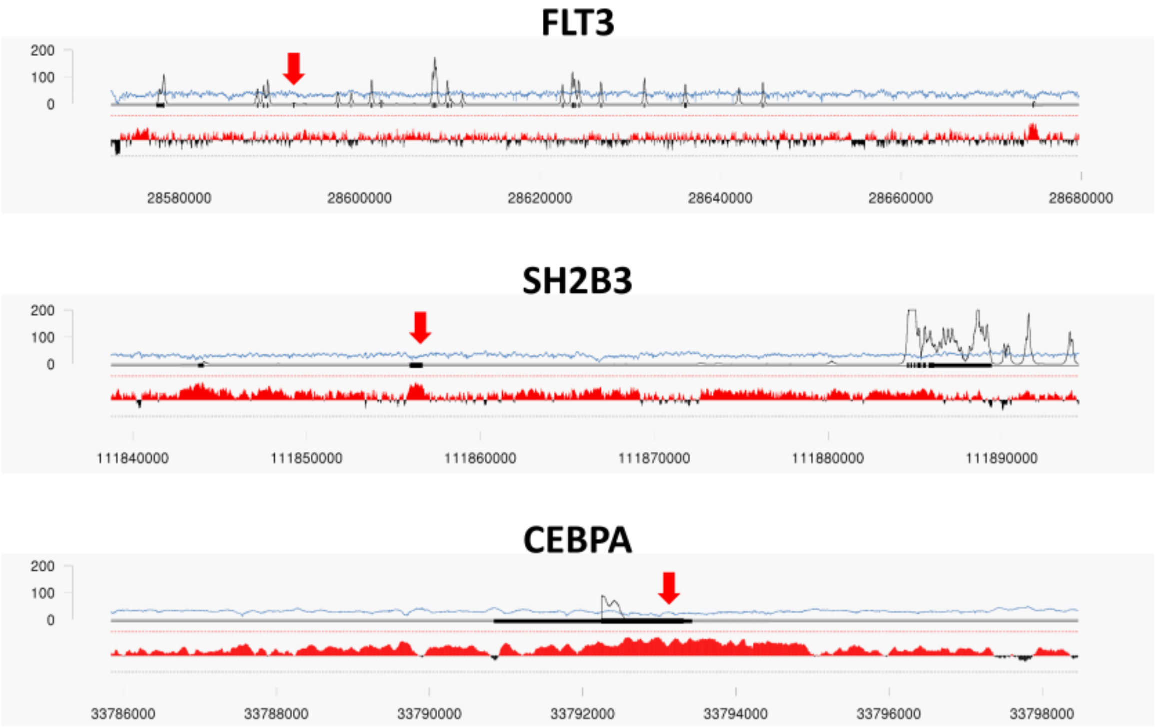

Supplementary Figure 6. WGS and WES coverage of three genes with P or LP SNVs or indels missed by WES. Plots show the WGS (blue) and WES (gray) coverage, averaged over all 78 germline samples, along the genes *FLT3*, *SH2B3*, and *CEBPA*. The gene model and GC content are depicted below the coverage graph, as in Supplementary Figure 1. Arrows indicate the location of P or LP SNVs/indels that were found by WGS but missed by WES. Each of these locations is well covered in WGS but systematically uncovered in WES.

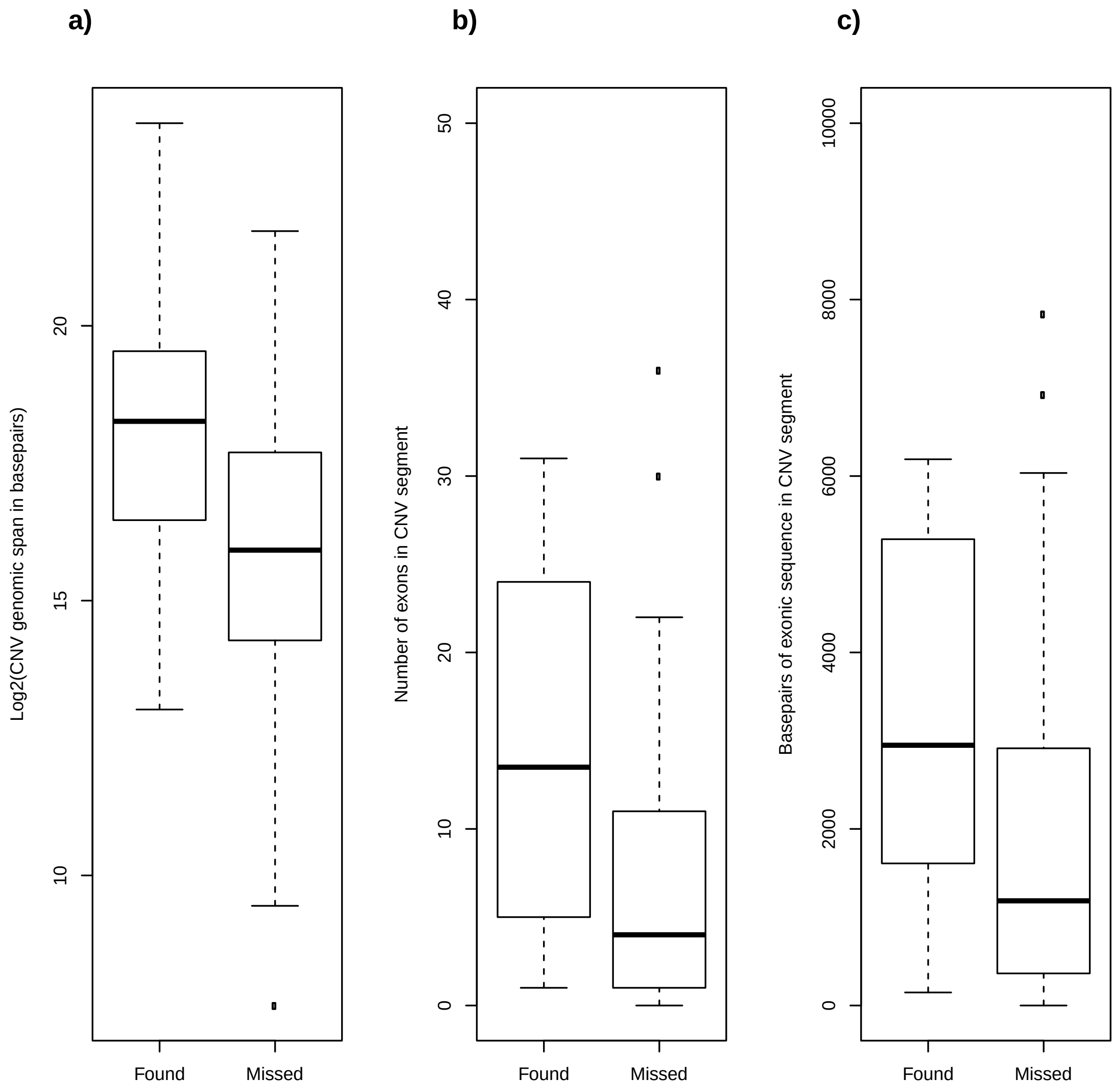

Supplementary Figure 7. Comparison of P/LP sub-arm CNAs detected or missed by WES CNA analysis. Each panel shows the distribution of CNAs detected by both WGS and WES (Found; n=18) or detected by WGS and missed by WES (Missed, n=36). The dark lines indicate median values. The boxes indicate the interquartile range (IQR). Whiskers extend to 1.5×*IQR* unless there are no outliers that exceed 1.5×*IQR*, in which case they are the minimum and maximum values. **a)** Distribution of CNA genome size (log2 of segment size in basepairs) **b)** Distribution of number of exons within CNAs **c)** Distribution of the number of basepairs of exonic regions covered by the CNA. Wilcox Rank Sum test showed significant differences in the distributions with p-values of p-value = 0.002253, 0.003404 and 0.005525 respectively.

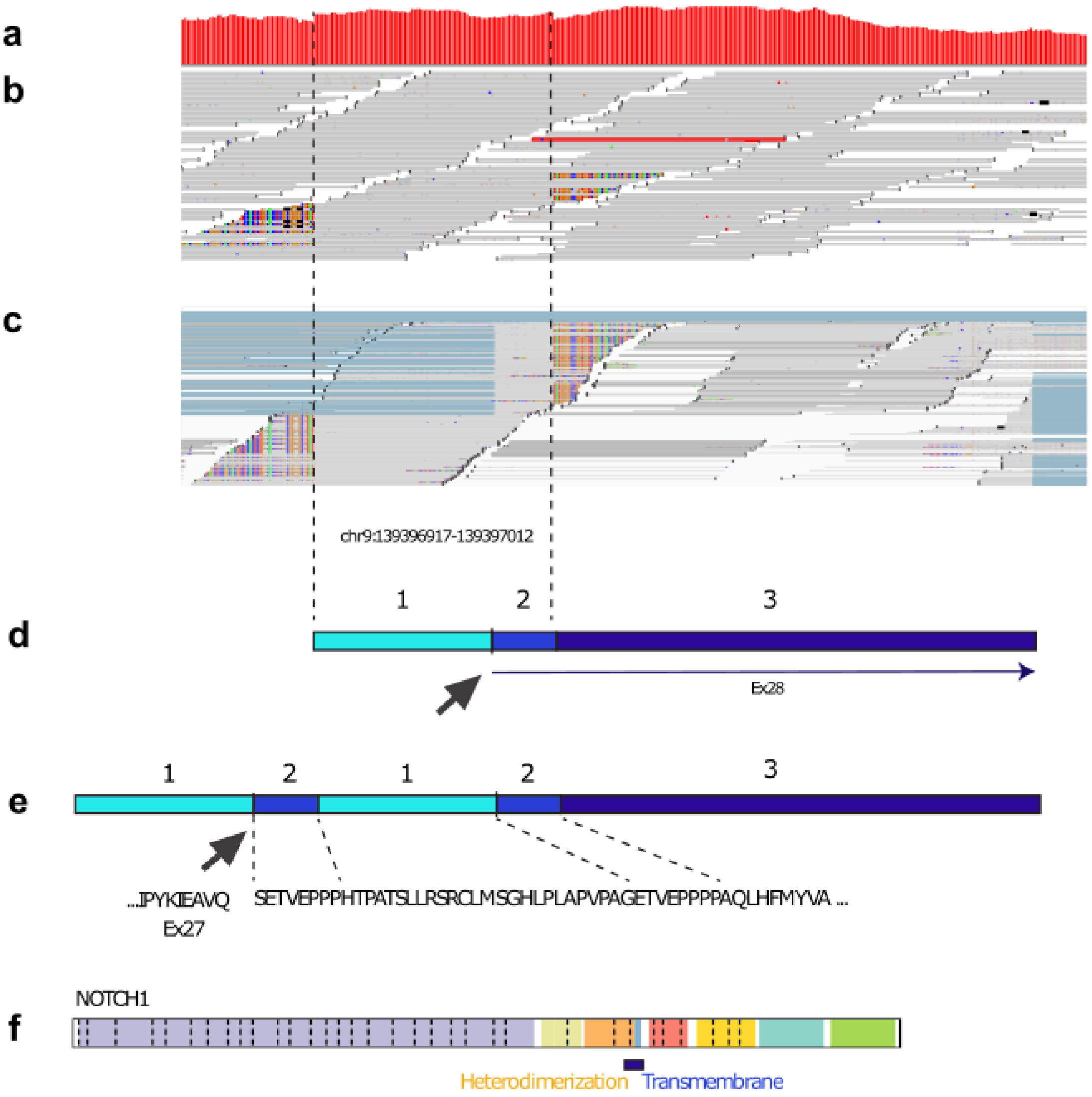

Supplementary Figure 8. *NOTCH1*-ITD in SJMLL002. (a) Whole genome coverage for a 362 bp region encompassing intron 27 and exon 28 of *NOTCH1* (NM_017617; chr9:139396703-139397065[hg19]); maximum coverage is 76X. Dotted lines throughout the figure indicate the position of a 96 bp duplication. **(b)** WGS showing normally mapped reads in grey with clipped basepairs in various other colors. CREST predicted a tandem duplication of chr9:139396917-139397012. **(c)** RNA-Seq showing reads as above, but additionally, canonical splicing from *NOTCH1* exon 27 (not shown) into *NOTCH1* exon 28 as light blue horizontal lines. Clipped reads are seen at the same positions as WGS and Cicero independently predicted the same duplication. **(d)** Schematic representation of the non-duplicated region. Region 1 is intron 27 sequence.

Region 2 is the start of exon 28; the position of the exon 28 splice acceptor is shown with a black arrow. The ITD is comprised of regions 1 and 2. Region 3 shows the non-duplicated portion of exon 28. **(e)** Schematic representation of the duplicated locus shows regions 1 and 2 are arrayed in tandem. Predicted protein translation based on Cicero's ITD contig is shown below. Exon 27 sequence splices into the upstream splice acceptor (black arrow). Although the ITD contains intronic sequence, the reading frame is preserved. **(f)** Schematic representation of *NOTCH1* from Protein Paint (https://pecan.stjude.org/#/proteinpaint/NOTCH1). The approximate position of the duplicated region is shown as a blue rectangle encompassing the distal end of the heterodimerization domain and the proximal end of the transmembrane domain. This region is known to undergo tandem duplication in T-ALL.

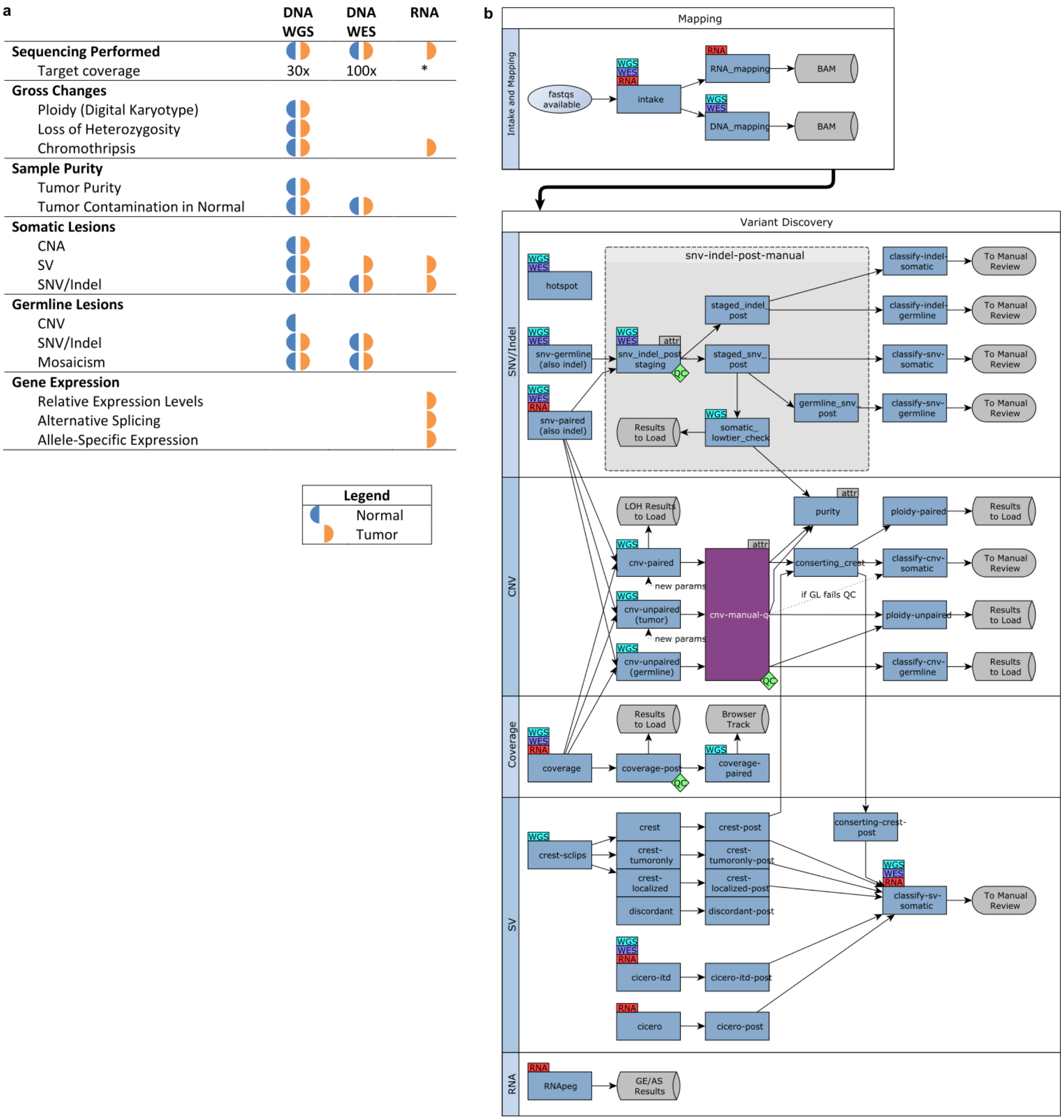

Supplementary Figure 9. Bioinformatics pipeline. (a) Tumor and normal DNA is sequenced using WGS to a target depth of 30x and WES to a target depth of 100x. Tumor RNA is also sequenced using RNA-Seq. Gross chromosomal changes, sample purity statistics, germline and somatic lesions, and gene expression information are each determined using one or more of the types of sequencing performed, as indicated by the semicircles. **(b)** A more detailed view of the pipeline used for mapping and variant discovery.

Supplementary Table 1. RT-PCR Ct Values

RT-PCR Ct values for **a)** *ETV6-RUNX1* and **b)** *KMT2A-MLLT3*. A relative increase of 3.2 Ct values between *GAPDH* and test gene indicates a log10-fold decrease in expression of the test gene.

a)

	Ct(*ETV6-RUNX1*)	Ct *GAPDH*
SJETV093	25.85	19.5
Pos control	21.65	15.15

b)

	Ct(*KMT2A-MLLT3*)	Ct *GAPDH*
SJMLL019	34.12	16.84
Pos control	23.33	17.45

SUPPLEMENTARY METHODS

WGS, WES and RNA-Seq Library Preparation and Sequencing

WGS libraries were constructed using the TruSeq DNA PCR-Free sample preparation kit (Illumina, Inc) following manufacturer's instructions. Briefly, 1 µg of genomic DNA was sheared by acoustic fragmentation using a Covaris E210 (Covaris, Woburn, MA) with the recommended settings for 350 bp fragments. The fragments were end-repaired, 3' adenylated and an indexed paired-end adapter ligated. The adapter-ligated library was purified using the sample purification beads provided in the kit prior to sequencing.

WES libraries were prepared using the TruSeq exome enrichment kit v1 (Illumina, Inc) per manufacturer's instructions, with some modifications. Briefly, 1 µg of genomic DNA was fragmented and end-repaired as above, then ligated with paired-end sequencing adaptors and amplified using 10 PCR cycles. At least 500 ng of each DNA library sample were pooled and hybridized with biotinylated oligo RNA baits corresponding to exome sequences for two-overnight incubations at 58ºC. Each overnight hybridization incubation was followed with binding to Streptavidin-conjugated Magnetic Beads, a washing step and an elution step. After the second hybridization and elution, the exome-enriched libraries were again amplified via a 10-cycle PCR. We optimized Illumina's TruSeq exome enrichment protocol by reducing the number of PCR cycles from 12 to 10 for both the pre- and post-enrichment libraries. We also noticed that the timing of the first

and second hybridization cleanups were critically important. These two modifications resulted in better library yield and lower sequencing read duplication rates.

RNA-Seq libraries were constructed as follows: Briefly, 0.1 – 1 µg of total RNA was ribo-zero gold treated for ribosomal RNA reduction - removing cytoplasmic ribosomal RNA (rRNA) and mitochondrial rRNA by hybridizing the rRNA to biotinylated capture probes. The ribosomal-reduced RNA was bead-purified then chemically fragmented and reverse transcribed with SuperScript III Reverse Transcriptase (Thermo Fisher Scientific) to generate complementary DNA (cDNA). The resulting cDNA fragments underwent end repair, adenylation of the 3' end and then ligation of adapters. The RNA-Seq libraries were PCR-enriched using 11 PCR cycles. We modified Illumina's standard TruSeq Stranded Total RNA protocol by reducing the number of PCR cycles from 15 to 11, resulting in better quality libraries and reduced sequencing read duplications. We also suggest that some degraded total RNA samples may not require fragmentation.

The resulting WGS, WES and RNA-Seq libraries were assessed for quality and size range using the Agilent 2100 BioAnalyzer (Agilent Technologies). Library quantity were determined using the Kapa NGS library quantification kit with Illumina library-specific primers and external standards (Kapa Biosystems) and analyzed on the Eco Real-Time PCR System (Illumina). Libraries were diluted to 2nM, denatured with sodium hydroxide and clustered either on the cBot (Illumina) using the TruSeq PE Cluster Kit v3-cBot-HS (Illumina) or directly on a HiSeq2500 instrument in Rapid mode (using the TruSeq Rapid PE Cluster kit) according to the manufacturer's instructions. Sequencing was performed

on HiSeq 2000 or HiSeq 2500 instruments with paired-end (2 x 101 bp) sequencing using TruSeq SBS Kit v3-HS or TruSeq Rapid SBS Kit (Illumina).

Computational Infrastructure

A dedicated compute and storage infrastructure was designed and implemented by the High Performance Computing Facility at St. Jude. One primary design goal was to isolate this resource as much as possible from external dependencies to minimize production impact and increase administrative control. The environment includes an 84 node HPC cluster with over 1,300 computing cores at a memory ratio of 8 GB system RAM per core, an InfiniBand FDR cluster interconnect in a fat tree topology, and an IBM GPFS parallel filesystem with redundant storage blocks yielding 500 TB of analysis workspace. The storage environment is also accessible on the internal institutional network using NAS protocols. This access is used by the sequencing lab (for receiving raw instrument run data), analyst workstations, and by various application and database servers providing support services. One such application service is the clinical genomics LIMS, which is integrated with the sequencers and the HPC environment to allow BCL conversion and demultiplexing to take place directly on the compute cluster before intake into the analysis pipeline. Integration with institutional identity management at all levels of the analysis infrastructure ensures that authorization to cluster and storage resources were controlled.

All analytical pipelines were run in an in-house software infrastructure consisting of our TARTAn API for Reproducible and Traceable Analyses and RAPTR (Rapid-Access Process Tracking and Reporting).

We developed TARTAn for managing all of the high performance computing runs and management of result files. With TARTAn, every run of an analysis pipeline takes place in an analysis-specific run directory which contains a standard set of files and subdirectories. The subdirectories are used to separate files into four classes that are managed differently: (1) symbolic links to input files, (2) intermediate files that are removed at the end of the run, (3) workspace files such as logs that are archived in a zip file at the end of the run, and (4) output files that are made read-only at the end of the run. The targets of the symbolic links from class (1) are output files from other runs, so that the full provenance of an output file can be determined. Output files are further linked from an index, which is organized by project, case, sequencing type, and file type, rather than by analysis run. The index allows easy and reliable determination of the most recent result for a particular case and analysis. In addition to the subdirectories, there are also files that store the environment during the run, from which the configuration and code version may be determined. Code and configuration releases were performed as TARTAn analyses, which allowed all previous versions of code to be retained at permanent file paths. This allowed us to deploy code and configuration changes while pipelines were still running; pipelines executing during the deployment would continue to use the previous version of code until they completed.

We developed the RAPTR database for tracking sequencing data and the processes run on it. The RAPTR database tracks information on every read group including sample, sequencing type, sequencer run and lane, and which BAM file(s) include mappings for the read group. It also tracks every analysis run that has been performed on a read group, a BAM file, or a pair of BAM files, including the current status of the run. The analysis runs in RAPTR correspond directly to the analysis runs in TARTAn, which facilitates status tracking.

Pipeline Automation Using RAPTR and TARTAn

We developed a class library that uses RAPTR and TARTAn to automate, fully or in part, the analysis runs that we perform. The class library has the ability to comb the database for samples that need to run through a particular analysis (i.e. all dependencies are satisfied and the analysis has not already run), setup the analysis run in TARTAn, and then execute the run. We employed cron to regularly invoke this process such that most of the analysis runs could be performed without requiring any user intervention. Almost none of the analyses that were performed were written to use TARTAn, so we developed wrappers that would create the TARTAn run, run the analysis in the intermediate file space, and then copy the final result files to output on completion. In this way, we could run a diverse set of analysis tools with little or no modifications to the underlying code.

Some analysis runs were not completely automated. For these, we wrote a program that uses RAPTR, and the symbolic links for input files in TARTAn, and the state of various

auditing files and directories in TARTAn, to automatically determine the status of the analysis run and which samples are included. Invocation of this script was incorporated into SOPs to ensure that the status of these partially-automated runs would remain up-to-date without significant manual effort.

Pipeline Validation using COLO-829

To evaluate the sensitivity and PPV of our pipeline, we made use of the well characterized COLO-829 cell lines, which provided a paired set of tumor and normal genetic material suitable for our combined somatic-germline analyses. We first compiled an extensive set of re-sequencing data for these cell lines, consisting of 14 WGS data sets (8 tumor, 6 normal) and 5 WES data sets (3 tumor, 2 normal). Using a previously published set of coding SNVs and indels from the initial COLO-829 genome publication [2], we assessed the sensitivity of our pipeline. All but 4 of the 292 variants initially reported by Pleasance, et al., were detected, yielding a baseline recall rate of 0.99. While no gold standard catalogue of COLO829 structural & copy number variants existed, we used extensive re-sequencing data and multiple internal (CONSERTING, CREST) and external [3,4] calling algorithms to construct a comprehensive list of 48 high confidence SV events. This result set was used in recurrent proficiency examinations to ensure that no pipeline modifications negatively impacted variant detection.

Limit of Detection Analysis

To assess the power to detect variants for our study design, we assumed a constant 100X for WES and 30X for WGS, and used binomial distribution to calculate the probability of observing >= 3 reads in one platform (as automatic detection) and observing >=1 reads in the other platform (as being observed for validation purpose), for underlying MAFs ranged from 0.01 to 0.5, which correspond to cancer cell fraction of 2% to 100% in diploid regions. To calculate the probability of one variant being detected and validated, we multiply the probability of automatic detection in one platform (i.e., >= 3 mut reads) and the probability of observing the mutant reads in the other platform (i.e., >=1 mut read), by assuming independence between samplings during WES and WGS sequencing. In addition, we performed re-sampling analysis to cross justify the above theoretical analysis, using *NRAS* G12D locus from case SJBALL021900 (with purity of 92%, and no sign of tumor in normal contamination, 53/110 in tumor WES, 19/45 in tumor WGS). For each predefined MAF, α, we sampled reads from tumor bam with probability α/0.92 and from normal bam with probability 1-α/0.92 with replacement. 100 reads were sampled from tumor for WES and 30 reads from tumor for WGS.

SUPPLEMENTARY NOTES

Supplementary Note 1: SNV/indel call rate in WES

As shown in Supplementary Data 6A-D, prior to quality filtering and cross-validation, there was a higher calling rate in WES than WGS for SNVs and more so for indels using the same threshold for variant detection [5,6]. Specifically, if we count only indels that had sufficient coverage in capture validation for ascertaining somatic mutation verification status as presented in Supplementary Data 6B, there are a total of 404 WES-only indels and 24 WGS-only indels. Of the 404 WES-only indels, only 14 (3.5% of 404) were of high quality while the vast majority (93.3%, 377 out of 404) were in highly repetitive regions of short tandem repeats (STR) or homopolymers of which nearly all (96.8%, 365/377) have low mutant allele fraction (MAF) of <0.1. Less than 4% of the WES-only indels were verified by custom capture. By contrast, of the 24 WGS-only indels, the majority (91.7%, 22/24) were of high quality and only a subset (33.3%, 8/24) were in repetitive regions. The majority (16 out of 24; 66.7%) of WGS-only indels were verified by custom capture even though many (15 out 24, 62.5%) had low MAF (<0.1).

The higher error rate of WES-only indels have been reported previously in a study that compared the indel genotype calls from WGS and WES in HapMap sample NA12878 [7]. Our study confirmed the previous observation as the low validation rate (<4%) of those WES-only indels; the large majority of which had low MAF and were predominantly located in highly repetitive regions.

Supplementary Note 2: Expression of DIP2C-PDGFRA fusion gene

As shown in Figure 2b, the *PDGFRA* amplification encompassed only the 3' end of *PDGFRA* and omitted the extracellular domains encoded by exons 1-9. Counting of wildtype and fusion junction reads from RNA-Seq showed 727 wild-type exon 9-10 junctions, implying expression of wild-type *PDGFRA* was higher than that of fusion gene whose RNA junction reads numbered 26 and 149 from *DIP2C* exon 1 and *PDGFRA* exons 10 and 11 respectively. This showed, surprisingly, that the expression of amplified region of *PDGFRA* was not strongly correlated with the level of DNA amplification. Further investigation showed that *DIP2C* has generally low expression in High Grade Glioma with FPKM of <14 in non-amplified PCGP samples (data is available at https://pecan.stjude.org/proteinpaint/DIP2C). Given that the weak *DIP2C* promoter drove fusion gene expression, we hypothesize that amplification was necessary to achieve a sufficient level of *DIP2C-PDGFRA* for oncogenic action. Similar rearrangements of *PDGFRA* including *KDR-PDGFRA* and *PDGFRA*Δ8, 9 that lack intact extracellular domains show *PDGFRA* kinase activation [8] and appear to be oncogenic even at relatively low expression levels. For example, Brennan et al. (2013) [9] used a >10% of total *PDGFRA* expression to call a sample positive for the rearrangement in their analysis of RNA-Seq generated from glioblastoma samples from The Cancer Genome Atlas (TCGA) project.

Supplementary Note 3: Heterogeneity of MYCN Amplifications in SJRB051

The inconsistency between *RB1* rearrangement and *MYCN* amplification implies that these events occurred at different times during tumor evolution. The DNA specimen for

this study was extracted from a different vial from PCGP (Supplementary Data 3, column K); however the bi-allelic *RB1* re-arrangements are present in both specimens, suggesting that disruption of *RB1* was an early event present in every tumor cell. In contrast, the *MYCN* amplification is likely to be a later event that is present in a subset of tumor cells. Given the high copy number and focal nature of the PCGP sample's *MYCN* amplification, episomal amplification via double minutes is a likely explanation [10]. Differing levels of oncogene amplification between cells of the same tumor has been previously reported in pediatric glioma,[11] and heterogeneity of *MYC*-bearing double-minutes has been previously reported in our study of medulloblastoma [3].

Supplementary Note 4: FX1R-BRAF fusion in SJLGG026

Neither WGS SV nor RNA-Seq SV detected the known *FXR1-BRAF* fusion as documented in Supplementary Data 7A. Manual inspection of the aligned WGS data recovered two breakpoint reads, insufficient for an SV call by our pipeline. The average WGS coverage in the tumor was 36.8X (Supplementary Data 3) and the coverage at the two breakpoint regions was 54X and 36X. Manual inspection of aligned RNA-Seq reads at expected positions of exon fusion recovered one fusion read, also insufficient for the pipeline to call. The fusion was initially discovered by WGS in our research project, PCGP. The PCGP WGS coverage for this tumor was 65X and the *BRAF* fusion was detected by 3 junction reads and the estimated MAF of 0.04 [12]. In RNA-Seq, FPKM for *FXR1* and *BRAF* is 7.4 and 6.8 respectively. Therefore, we would conclude that the failure to identify the fusion in WGS and RNA-Seq was caused by the low MAF (0.04) of the fusion in a sample with low tumor purity.

Supplementary Note 5: Pathologic and likely pathologic mutations not detected by WES

A total of seven pathologic or likely pathologic somatic SNVs/indels were discovered by WGS alone. Of those seven, two (*FLT3* and *SH2B3*) had no support in WES, and five had insufficient support in WES for de novo detection (Supplementary Data 7C). We manually inspected each of the seven to determine the cause for non-detection in WES. Four out of the seven samples also had whole exome sequencing from various research studies. For these four, we combined the reads from the clinical and research experiments and ran the resulting data through the analytical pipeline to determine if additional coverage would allow the variants to be detected by WES. The results are given in Supplementary Data 7C. In brief, three of the seven were in regions of systematically low coverage and would therefore be unlikely to be recovered by additional coverage (one had additional reads, and it was not detected with additional coverage). One appeared to be caused by poor capture of the indel-harboring fragments and would also be unlikely to be recovered by additional sequencing. The other three showed no signs of systematic WES-related problems and were all recovered with additional reads.

Supplementary Note 6: Additional Analysis on Gene Fusions Detected only by WGS

Three gene fusions, *KMT2A-MLLT3* in SJMLL019, *ETV6-RUNX1* in SJETV093 and *KIAA1549-BRAF* in SJLGG020, were detected only by WGS. The RNA-Seq coverage of SJLGG020, SJMLL019 and SJETV093 had 43-47% of the exons with >20X coverage

while the average of the entire cohort was 47% (Supplementary Data 4). Therefore, the RNA-Seq coverage of all three samples was within the normal range for the study.

We also examined the expression level of the two fusion partner genes in these three cases (Supplementary Data 7A). Specifically, *KIAA1549* was expressed at a low level in SJLGG020 (FPKM of 4.30 and 10.0145 for *KIAA1549* and *BRAF* respectively). Low expression of *KIAA154*, whose promoter drives the fusion, provides a good explanation for the absence of detectable *KIAA1549-BRAF* fusions transcripts in RNA-Seq, particularly as an estimated 70% of *KIAA1549* expression would come from the wildtype haplotype based on estimated tumor purity from WGS data (Supplementary Data 3). Expression of *KMT2A-MLLT3* in SJMLL019 (FPKM of 7.93 and 2.64 respectively) shows low expression of *MLLT3*. Expression of *ETV6-RUNX1* in SJETV093 (FPKM of 27.53 and 25.02 respectively) were not as low as the previous two examples; however the reverse transcriptase PCR (RT-PCR) experiment described below shows that the fusion transcript itself was expressed at a low level. We performed reverse transcriptase PCR (RT-PCR) to quantify expression levels of *KMT2A-MLLT3* in SJMLL019 and *ETV6-RUNX1* in SJETV093. Using standard procedures, we calculated *ETV6-RUNX1* and *KMT2A-MLLT3* expression relative to the housekeeping gene, *GAPDH* in samples SJETV093 and SJMLL019 respectively as well as in positive control cell lines. The *ETV6-RUNX1* fusion expression was approximately two logs lower than the level of *GAPDH* in both the control cell line and in SJETV093. *KMT2A-MLLT3* showed a similar pattern with the expression of the fusion in the control cell line 1.5-2.0 logs lower than *GAPDH*. In SJMLL019, the expression of the fusion was approximately 5 logs lower

than the expression of *GAPDH* (or 3 logs lower than the cell line fusion expression level). For reference, common fusions assayed on our lab, including *RUNX1-RUNXT1*, *TCF3-PBX1* and *BCR-ABL,* are typically less than one log-fold lower than *GAPDH*. Cycle threshold (Ct) values are shown in Supplementary Table 1. In summary, all of the fusions that RNA-Seq missed were expressed at low levels in tumor samples.

Supplementary Note 7: Comparison of validation statistics on variants that passed the cross-validation filter with unfiltered variants

The cross-validation filter, which integrates variant calls from multiple platforms, is a critical process to ensure high accuracy of variant analysis in our clinical pipeline. Using exonic SNVs as an example, we show that cross-validation filtering improves PPV while maintaining a high sensitivity.

The PPV and sensitivity for exonic SNVs that pass cross-validation is 99% and 94%, respectively as shown in Figure 3 based on variant count of actual positives (n=695), predicted positives (n=662) and true positives (n=653) presented in Supplementary Data 6D. The summary variant counts were derived from raw variant data presented in Supplementary Data 6A-C which could also be used for calculating statistics on unfiltered variants. The following process can be used to calculate PPV and sensitivity for unfiltered variants combining WGS and WES data. By selecting variants detected by WGS or WES (filter column B "Platform" by selecting WGS_WES, WGS_ONLY, WES_ONLY) for unfiltered variants (select all in column C "Pipeline") that are covered by custom capture (In column A, de-select "UNCOVERED"), there are a total of 764

“predicted positive” variants that can be evaluated for their accuracy using the custom capture data. By selecting “SOMATIC” and “SOMATIC*” in column A, there are a total of 661 variants which are the true positive variants. Therefore, unfiltered variants detected by WGS or WES would have a PPV of 87% (661/764) with a sensitivity of 95% (661/695). This shows that the cross-validation filter implemented in our clinical pipeline is highly effective in improving PPV by 12% while maintaining a high sensitivity (94% using filtered variants compared to 95% using unfiltered variants).

Supplementary References

1. Van Allen, E. M. *et al.* Whole-exome sequencing and clinical interpretation of formalin-fixed, paraffin-embedded tumor samples to guide precision cancer medicine. *Nat. Med.* **20,** 682–688 (2014).
2. Li, H. & Durbin, R. Fast and accurate long-read alignment with Burrows-Wheeler transform. *Bioinformatics* **26,** 589–595 (2010).
3. Rausch, T. *et al.* Genome sequencing of pediatric medulloblastoma links catastrophic DNA rearrangements with TP53 mutations. *Cell* **148,** 59–71 (2012).
4. Chong, Z. *et al.* novoBreak: local assembly for breakpoint detection in cancer genomes. *Nat. Methods* **14,** 65–67 (2017).
5. Zhang, J. *et al.* The genetic basis of early T-cell precursor acute lymphoblastic leukaemia. *Nature* **481,** 157–163 (2012).
6. Zhou, W. *et al.* ClinSeK: a targeted variant characterization framework for clinical sequencing. *Genome Med.* **7,** 34 (2015).
7. Fang, H. *et al.* Reducing INDEL calling errors in whole genome and exome sequencing data. *Genome Med.* **6,** 89 (2014).
8. Ozawa, T. *et al.* PDGFRA gene rearrangements are frequent genetic events in PDGFRA-amplified glioblastomas. *Genes Dev.* **24,** 2205–2218 (2010).
9. Brennan, C. W. *et al.* The somatic genomic landscape of glioblastoma. *Cell* **155,** 462–477 (2013).
10. VanDevanter, D. R., Piaskowski, V. D., Casper, J. T., Douglass, E. C. & Von Hoff, D. D. Ability of circular extrachromosomal DNA molecules to carry amplified MYCN proto-oncogenes in human neuroblastomas in vivo. *J. Natl. Cancer Inst.* **82,** 1815–1821 (1990).

11. Paugh, B. S. *et al.* Genome-wide analyses identify recurrent amplifications of receptor tyrosine kinases and cell-cycle regulatory genes in diffuse intrinsic pontine glioma. *J. Clin. Oncol. Off. J. Am. Soc. Clin. Oncol.* **29,** 3999–4006 (2011).

12. Zhang, J. *et al.* Whole-genome sequencing identifies genetic alterations in pediatric low-grade gliomas. *Nat. Genet.* **45,** 602–612 (2013).
