# Supplementary material for: Clinical cancer genomic profiling by three-platform sequencing of whole genome, whole exome and transcriptome
Source: Nat Commun. 2018 Sep 27;9:3962. doi: 10.1038/s41467-018-06485-7 (PMC6160438; doi:10.1038/s41467-018-06485-7)
Supplement: Supplementary file 3 — Description of Additional Supplementary Files [file 41467_2018_6485_MOESM3_ESM.docx]

**Description of Additional Supplementary Files**

File Name: Supplementary Data 1

Description: Cases. A listing of the 78 cases used in this study. The cancer subtype codes in column C are defined in column D. The tumor and germline sample names are listed in columns E and F and are used throughout the supplementary data. In the tissue type columns, G and H, bone marrow is abbreviated BM and peripheral blood is abbreviated PB.

File Name: Supplementary Data 2

Description: Previous Findings. The 86 findings from previous diagnostic testing are listed, along with the method of prior detection. In column D for abnormal findings, internal tandem duplication is abbreviated ITD.

File Name: Supplementary Data 3

Description: Samples. Detailed information about each sample is given. Column B indicates whether the case is one of the 18 cases that had all of the data necessary to be included in the sensitivity and PPV analysis. Columns D and O indicate whether the sample used in this study is the same as the sample previously used in the Pediatric Cancer Genome Project (PCGP); here, “Same” indicates that the sample used in this study was also previously used in PCGP, “Different” indicates the case was in PCGP but a different sample was used in this study, and “No” indicates the case was not part of PCGP. In some cases, the PCGP coverage values are listed as “NA”; this indicates that that type of sequencing was not performed on that sample in PCGP.

File Name: Supplementary Data 4

Description: Mapping Statistics. Mapping and coverage statistics for each sample/sequencing type are given. Columns J-M indicate the proportion of coding exons with average coverage exceeding the threshold indicated in the header.

File Name: Supplementary Data 5

Description: Germline Cancer Genes. Here we list the 61 genes used for germline reporting, along with our selection rationale.

File Name: Supplementary Data 6

Description: Validation Data. Data used for calculating sensitivity and PPV are presented in tables A-C (for SNV, indel, and SV) and summarized in table D. For tables A-C, many of the columns are self-explanatory, and the others are described below:

| Column | Description |
| --- | --- |
| CaptureVal Result | Variant status based on capture validation:   - SOMATIC: true somatic variant - GERMLINE: true variant, but of germline, not somatic, origin - WILDTYPE: not a variant - UNCOVERED: capture validation had insufficient coverage to make a determination. |
| Platform | Platform that was able to discover the variant: WGS_ONLY, WES_ONLY, or WGS_WES for both. |
| Pipeline | Whether or not the variant passed the validation filter in the clinical pipeline. |
| PCGP vs Clinical | Whether the variant was called in the CLINICAL or PCGP analysis or BOTH. For the purposes of this column, variants originally called in clinical but labeled as FailFilter are counted as called in CLINICAL. |
| Pos | Position (left endpoint for indels) |
| OrtA (6C only) | Which side of the breakpoint is moved proximal to locus B by the structural variation. (+) indicates the left and (-) indicates the right. |
| OrtB (6C only) | Which side of the breakpoint is moved proximal to locus A by the structural variation. (+) indicates the right and (-) indicates the left. |
| #Mut_T | Number of reads supporting the variant in tumor |
| #Tot_T | Total coverage at the site in tumor |
| #Mut_N | Number of reads supporting the variant in germline |
| #Tot_N | Total coverage at the site in germline |
| Flanking | Flanking sequence. Reference/variant allele are inside square brackets. |

File Name: Supplementary Data 7

Description: Pathologic Findings. Table A lists all pathologic, likely pathologic, and VUS findings from this study and previous study. Column F indicates the classification as either pathologic (P), likely pathologic (LP) or variant of uncertain significance (U). Columns H-N indicate the status of variant detection by various algorithms and platforms. The values in these columns can be interpreted as follows:

| Value | Meaning |
| --- | --- |
| Y | Variant detected by this method. |
| Rescue | Variant was "rescued" by manual inspection. Rescued variants were not included in standalone platform assessments. |
| Complex | Complex finding was the result of manual interpretation of multiple calls. |
| Suggestive | WES CNV provided evidence for a fusion, but additional evidence would be required to report. |
| N | WES CNV did not provide evidence for a variant. |
| (blank) | Method did not show evidence for a variant. |

Table B contains additional details including read count information for SNV and indel calls.

Table C contains the results of our analysis of the somatic SNVs and indels that were detected by three-platform sequencing but missed by WES alone.

File Name: Supplementary Data 8

Description: Sub-arm CNV Comparison. This table lists the CNVs that are smaller than arm level and compares detection and reporting of these sites in CONSERTING using WGS and Sequenza using WES.

File Name: Supplementary Data 9

Description: Somatic Cancer Genes. Here we list the known cancer genes used in pathogenicity assessment.
